# A Comparative Assessment of Flaxseed (*Linum usitatissimum* L.) and Chia Seed (*Salvia hispanica* L.) in Modulating Fecal Microbiota Composition and Function In Vitro

**DOI:** 10.1002/fsn3.70243

**Published:** 2025-05-02

**Authors:** Seda Arioglu‐Tuncil

**Affiliations:** ^1^ Department of Nutrition and Dietetics Necmettin Erbakan University Konya Türkiye

**Keywords:** 16S rRNA, chia seed, colonic microbiota, fecal fermentation, flaxseed, seeds, short‐chain fatty acids

## Abstract

Flaxseed ( *Linum usitatissimum* L.) and chia seed ( *Salvia hispanica* L.) have become increasingly popular in the design of various functional food products. However, information on their functional properties is scarce. The aim of this study is to comparatively evaluate the effects of the dietary fibers (DFs) of flaxseed and chia seed on colonic microbiota composition and metabolic outputs. The neutral and acidic monosaccharide compositions of DFs of flaxseed and chia seed were determined using gas chromatography/mass spectrometry (GC/MS) and spectrophotometer, respectively. Next, in vitro fecal fermentation assays were applied, and samples were collected at different time points for short‐chain fatty acids (SCFA) measurements using GC, and fecal microbiota changes before and after fermentation were evaluated through 16S rRNA sequencing. The results revealed that DFs of flaxseed were dominated by xylose and uronic acid moieties, while that of chia seed was dominated by glucose units, indicating that their DFs are structurally different. Higher SCFA generations were observed in the case of flaxseed, suggesting that flaxseed DFs are more readily fermentable by gut microbiota. Flaxseed and chia seed DFs differentially impacted the microbiota compositions at the OTU level; for example, significant increases in the relative abundances of *Acidaminococcaceae* and *Bacteroides stercoris* related OTUs, which are known to be propionate producers, were observed in the case of flaxseed, but not chia seed. Interestingly, flaxseed, but not chia seed, DFs suppressed the growth of some pathogenic bacteria. Overall, this study suggests that the functionality of flaxseed and chia seed DFs in relation to colonic microbiota may differ, with flaxseed being more readily fermented and potentially promoting beneficial microbes to a greater extent. Thus, flaxseed could hold promise for developing functional food recipes aimed at supporting colonic health.

## Graphical abstract

Chia seed and flaxseed dietary fibers differ in composition. Chia seed and flaxseed differentially impact colonic microbiota composition and metabolic outputs (graphical abstract was created with BioRender.com ‐ https://BioRender.com/c31g929).

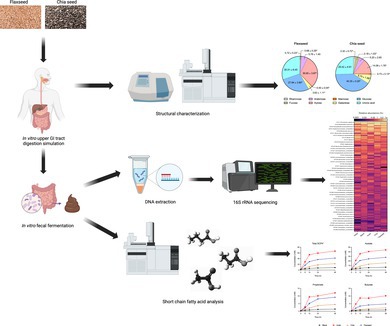

## 1 Introduction

The growing global population, along with the increasing depletion of food resources and a dramatic rise in modern diseases such as cancer, diabetes, and obesity, among others, has prompted food authorities to prioritize the promotion of more sustainable diets for societies. A sustainable diet should contain nutrition‐dense ingredients while incorporating environmentally sustainable food alternatives, minimizing ecological impact, and promoting long‐term human health. Moreover, food ingredients used to formulate a sustainable diet need to be culturally acceptable. In recent years, plant‐based foods have received considerable attention due to their rich nutrient contents, health benefits, being environmentally sustainable, and shaping the dietary habits of societies.

Seeds are an important class of plant‐based foods as they are often the parts of plants with the highest concentration of nutrients. For instance, chia seed ( *Salvia hispanica* L.) and flaxseed ( *Linum usitatissimum* L.) are becoming prominent within plant‐based food alternatives since they are rich in fiber, fatty acids, and antioxidants. Recent studies have shown that flaxseed and chia seed offer a variety of health benefits. Flaxseed has been recognized for its antioxidant and antidiabetic properties, demonstrating positive effects in the treatment and control of obesity, cardiovascular diseases, neurological conditions, various cancer types, and gastrointestinal disorders (Boueri et al. 2015; Dzuvor et al. 2018; Parikh et al. 2019; Kauser et al. 2024; Tian et al. 2025; Kunutsor et al. 2025). Similarly, chia seed has been reported to exhibit anti‐tumor, antioxidant, and antidiabetic properties (Enes et al. 2020; Wang et al. 2024; Cisternas et al. 2024). The majority of health benefits of chia seed and flaxseed are attributed to their high dietary fiber contents and other bioactive compounds such as phenolic compounds (Luo et al. 2018).

Per 100 g, chia seed has been reported to contain 32.16 g of lipids, 18.18 g of protein, and approximately 30–34 g of dietary fiber, of which the insoluble fraction (IDF) accounts for approximately 85%–93%, while soluble dietary fiber (SDF) makes up approximately 7%–15% (Ciftci et al. 2012). Similarly, 100 g of flaxseed is reported to contain 20.3 g of protein, 37.1 g of fat, and 24.5 g of fiber (Kajla et al. 2015). These high dietary fiber contents make flaxseed and chia seed excellent sources of dietary fiber, highlighting their potential for use in developing high‐fiber diets aimed at promoting a healthy lifestyle.

Dietary fibers are known as dietary compounds (mainly in carbohydrate structure) that are resistant to enzymes secreted in the human body and reach the colon. Microorganisms residing in the colon, known as colonic microbiota, use dietary fibers as a primary energy source by fermenting them partially or completely (Sekirov et al. 2010). It has been well known that colonic microbiota plays an important role in host health and disease. For example, a dysbiotic colonic microbiota composition—also referred to as a disruption of the microbial community—can significantly impact the occurrence and progression of various diseases, such as cancer, inflammatory bowel diseases, malnutrition, malabsorption, and obesity (Sekirov et al. 2010). Therefore, it is essential to maintain a healthy gut microbiota. Recent studies have emphasized the importance of the consumption of a high dietary fiber diet that contains different types of dietary fibers coming from various food sources, as it helps to sustain a healthy colonic microbiota composition by supplying the necessary energy to the members of this community. As a result of the fermentation of dietary fibers, various metabolites, mainly short‐chain fatty acids (SCFA), are produced by colonic microbiota, which have many local and systemic effects on the human body.

Several studies have investigated the effects of chia seeds and flaxseeds on gut microbiota, with the latter being studied in greater depth. For example, the impact of chia consumption on rats was evaluated, showing that a chia‐enriched diet did not affect the diversity of intestinal bacteria but led to increased richness in the intestinal microbiota, compared to the standard diet (Mishima et al. 2022). Additionally, chia consumption led to an increase in the abundance of the genus *Roseburia* (Mishima et al. 2022). Furthermore, the effect of chia mucilage on the metabolic activity of the human gut microbiota was examined using Simgi, a dynamic gastrointestinal model. The study revealed that chia mucilage did not alter the physical properties of the intestine but may influence bacterial growth and metabolic activity, particularly affecting the growth of *Enterococcus* spp. and *Lactobacillus* spp. (Tamargo et al. 2018). Similarly, the impact of whole milled flaxseed and partially defatted flaxseed press cake on the gut microbiota and the release of bioactive compounds was explored in vitro, revealing that *Bacteroidetes* emerged as the predominant phylum (Kleigrewe et al. 2022). Moreover, the effect of flaxseed polysaccharides on metabolic syndromes was investigated in high‐fat diet‐fed mice, demonstrating a reduction in fasting serum glucose, triglycerides, and cholesterol levels, and enhancing the proportions of *Akkermansia* and *Bifidobacterium* (Yang et al. 2020). Further research on flaxseed in elderly individuals with functional constipation indicated improvements in gut health (Ma et al. 2022). A study also examined whether flaxseed alters gut health in healthy mice, showing that flaxseed improved intestinal barrier integrity, and increased *Prevotella* spp. and reduced *Akkermansia muciniphila* abundance was observed (Power et al. 2016). Finally, the digestion and fermentation of flaxseed polysaccharides in simulated saliva, gastric, and small intestine conditions were assessed, revealing that flaxseed polysaccharides modulated gut microbiota composition and altered the *Firmicutes/Bacteroidetes* ratio (Zhou et al. 2020). Despite extensive research on each, their effects on colonic microbiota composition and short‐chain fatty acid (SCFA) production have not been comparatively assessed. Thus, the aim of this study was to comparatively assess the impact of the dietary fiber components of chia and flaxseed on colonic microbiota composition and function. To achieve this, the dietary fiber profiles of chia and flaxseed were analyzed, followed by in vitro fecal fermentation assays to evaluate their effects on microbiota composition and the production of microbial SCFAs.

## 2 Materials and Methods

Chia seed (black) and flaxseed were purchased from a local market in Konya, Türkiye. The samples were first chopped to coarse particles using a blender, followed by hexane treatment for 30 min (seed to hexane ratio was 1:3 (w/v)) to remove fat. Defatted samples were then ground using a coffee grinder to reduce the particle size of the seeds. The samples were then stored at –20°C until further analysis.

### 2.1 Simulation of Upper Gastrointestinal Tract Digestion of Chia Seed and Flaxseed

Chia seed and flaxseed were subjected to enzymatic treatment with pepsin and pancreatin, as previously described (Tuncil et al. 2018) to hydrolyze and eliminate digestible components that could be degraded by human enzymes. Briefly, 6.25 g of ground samples were suspended in 300 mL of water and placed on a heating bath circulator with a magnetic stirrer working at 37°C. After equilibration, the pH of the suspension was adjusted to 2.5 using 1 M HCl, followed by the addition of 2.5 mL of 100 mg/mL pepsin solution (Sigma‐Aldrich #P‐7000) dissolved in 50 mM HCl, and incubation for 30 min under constant stirring. Next, 12.5 mL of 0.1 M sodium maleate buffer was added, and the pH of the suspension was adjusted to 6.9 using 1 M sodium bicarbonate. 12.5 mL of 125 mg/mL pancreatin (Sigma‐Aldrich #P‐7545) dissolved in 0.1 M sodium maleate buffer was included, and the samples were incubated for another 6 h at 37°C under constant stirring. Three volumes of cold ethanol were added to the mixture, which were then kept at 4°C overnight to precipitate the dietary fibers. Precipitated dietary fibers were recovered through filtration (Whatmann paper #1), washed with 80% ethanol twice, and dried in a vacuum oven at 40°C for at least 2 h. The dried samples were then ground using a coffee grinder and stored at −20°C until further analysis.

### 2.2 Determining the Neutral and Acidic Monosaccharide Compositions of DFs of Chia Seed and Flaxseed

The neutral and acidic monosaccharide compositions of the samples were analyzed using gas chromatography coupled with mass spectrometry (GC/MS) and spectrophotometry, respectively, as previously described (Arioglu‐Tuncil et al. 2025). Briefly, 5 mg of sample was first subjected to hydrolysis using 72% sulfuric acid (250 mL) for 1 h at room temperature. 2.75 μL of water was then added to the samples, and they were subjected to further hydrolysis at 100°C for 3 h. At the end of the hydrolysis step, the samples were neutralized using 100 μL of undiluted ammonia. To analyze the neutral monosaccharide composition, 100 μL of the hydrolyzed sample was placed into a clean tube and dried under nitrogen. Then, the samples were subjected to the reduction and acetylation steps according to the procedure proposed by Pettolino et al. (2012). Acetylated samples were analyzed using GC/MS (6890 A, Agilent Technologies Inc., California, USA) coupled with a capillary column (RTX‐2330, Restek Corp., Bellefonte, PA, USA). The GC/MS conditions were as follows: Injection temperature of 240°C, injection volume of 0.2 μL, colon flow rate of 1.14 mL/min (helium was used as the carrier gas), colon initial temperature of 160°C where the samples were held for 7.15 min, and the temperature was then increased to 220°C with an increase of 4°C/min (hold for 4.10 min), and then further increased to 240°C with an increase of 2.90°C/min (hold for 5.15 min) and finally increased to 260°C with an increase of 10.80°C/min (hold for 5.10 min). Ion source temperature was set to 260°C.

Uronic acid contents of the chia seed and flaxseed were determined by adding 100 μL of NaCl/boric acid solution to 100 μL of the hydrolyzed sample, which had been transferred to clean tubes beforehand. Then, 1.6 mL of 18 M sulfuric acid was added to the tubes, followed by incubation at 70°C for 40 min. After the incubation period, 100 μL 3,5‐dimethylphenol (100 mg diluted to 100 mL with glacial acetic acid) was added to the tubes. The samples were then analyzed by a spectrophotometer at 400 nm and 450 nm, 10–25 min after the addition of 3,5‐dimethylphenol, as reported by AACCI (1999—Approved Methods of Analysis, Method #32–25.01). All the samples were analyzed in duplicate.

### 2.3 In Vitro Fecal Fermentation

An anaerobic chamber (BACTRON300 Anaerobic Chamber; Shel Lab, Cornelius, OR) containing 90% N_2_, 5% H_2_, and 5% CO_2_ was used to conduct an in vitro fecal fermentation assay, according to the method described by Tuncil et al. (2018). Briefly, 25 mL balch test tubes containing 50 mg of the sample were prepared for selected time points (0, 6, 12, 24 and 48 h), along with inulin as a positive control and a substrate‐free blank sample as a negative control. The tubes were then autoclaved at 121°C for 20 min, followed by being placed in the anaerobic chamber overnight. The following day, 4 mL of sterilized and reduced carbonate‐phosphate buffer was added to each tube. Fecal samples of three healthy donors were collected in fecal sample collection containers with ice. The following criteria were used to select the healthy donors for fecal sampling: (1) adherence to their routine diets; (2) no use of antibiotics in the past 3 months; (3) a body mass index between 18.5 and 25 kg/m^2^. The fecal samples were promptly placed in the anaerobic chamber and processed within 2 h of collection. Carbonate‐phosphate buffer with a ratio of 1:3 (feces: buffer, w/v) was used to homogenize the fecal materials. Then, four layers of cheesecloth were used to filter fecal material, followed by pooling the filtrates in equal volumes. The pooled fecal slurry was transferred (1 mL) into each balch tube, which were then closed with butyl rubber stoppers (Chemglass Life Sciences # CLS‐4209‐14), hermetically sealed with aluminum seals (Chemglass Life Sciences #CLS‐4209‐12), and incubated at 37°C in a shaking incubator at 150 rpm. All fermentation assays were conducted in triplicate.

At selected time points of fermentation, two aliquots were taken from each tube: one for DNA extraction (1.6 mL) and the other for was SCFA analysis (0.8 mL). To analyze SCFAs, the internal standard was prepared by mixing 157.5 μL of 4‐methylvaleric acid, 1.47 mL of 85% phosphoric acid, and 39 mg of copper sulfate pentahydrate, with ultrapure water added to reach a final volume of 25 mL. The samples were kept at −20°C until further use. The protocol for collecting and using human fecal materials were approved by the Scientific Research Ethics Committee of Health Sciences of Necmettin Erbakan University (application # 19778; approval # 2024/782). All the procedures followed were under “The Regulation on the Scientific Research Ethics Committee of Health Sciences” of Necmettin Erbakan University in accordance with the standards of the Declaration of Helsinki. Before conducting the study, a written consent form was collected from the donors.

### 2.4 Short‐Chain Fatty Acids (SCFAs) Analysis

A gas chromatography (GC/FID, GC‐2030, Shimadzu) coupled with a fused silica capillary column (Restek Stabilwax Column) was used to analyze and quantify the microbial SCFAs, namely acetate, propionate, and butyrate—generated during fermentation, following the method described by Tuncil et al. (2018). Briefly, the samples collected for SCFA analysis were removed from the freezer and thawed before being centrifuged at 13,000 rpm at 4°C for 10 min.

2 μL volume of the supernatant was then injected into the GC through the autosampler unit. The following operating conditions were used for GC: injector temperature: 230°C; injection volume 2 μL (split ratio: 1: 20); oven temperature was set to 100°C. This temperature was held for 1 min and then ramped at 8°C/min to 200°C, held for 5.50 min; FID temperature was at 250°C. Acetate, propionate, and butyrate purchased from Sigma‐Aldrich (St. Louis, MO, USA) were used to prepare external standards.

### 2.5 DNA Extraction, 16S rRNA Sequencing, Sequence Processing, and Community Analysis

DNA extraction was performed using the samples collected at 0 and 24 h of fermentation. The samples were initially thawed at room temperature, then centrifuged at 13,000 rpm for 10 min at 4°C. The supernatant was discarded, and DNA extraction was conducted using the pellet recovered after centrifugation following the chemoenzymatic lysis approach outlined by Ferrera et al. (2010) and modified by Lindemann et al. (2013) with the bead‐beating phenol‐chloroform procedure reported previously for fecal samples by Mackenzie et al. (2015). The detailed description of the method was provided by (Tuncil et al. 2018). The 515F and 806R primers targeting the V4 region of the 16S rRNA gene were used for amplification. The details of the amplification procedure are given at the Earth Microbiome Project website (https://earthmicrobiome.org/protocols‐and‐standards/16s/). The samples were barcoded using Tru‐Seq barcoding primers and sequenced on an Illumina MiSeq run with 2 × 250 cycles and V3 chemistry (Illumina Inc., San Diego, CA, USA) at the Teknogen BioEngineering Company (Ankara, Türkiye). Sequences were processed using Mothur version 1.48.0 according to the Mothur MiSeq SOP (https://mothur.org/wiki/miseq_sop/) (Kozich et al. 2013; Schloss et al. 2009).

### 2.6 Statistical Analysis

GraphPad Prism version 10 for Mac OS X (GraphPad Software Inc., La Jolla, CA, USA) was used for data visualization and statistical analysis. The results are shown as mean ± standard error. Except for the monosaccharide compositional analysis, where the statistical differences between monosaccharide moieties of two samples were determined using two‐tailed student's *t*‐test at α = 0.05, significant differences between the samples and controls were assessed using analysis of variance (ANOVA) at a significance level of α = 0.05. To identify statistically significant mean differences, Tukey's multiple comparison test was performed at the α = 0.05 level. The analysis of molecular variance (AMOVA) test implemented in Mothur was used to assess statistical differences in β‐diversity indexes.

## 3 Results and Discussion

### 3.1 Monosaccharide Compositions of Chia Seed and Flaxseed Dietary Fibers

Flaxseed and chia seed are known to be excellent sources of dietary fibers; the former's dietary fiber content was reported to range from 36.7% to 46.8% (Singh et al. 2011), while the dietary fiber content of the latter can go up to 56.46% (Din et al. 2021). Although dietary fibers of both flaxseed and chia seed were reported to be composed of lignin, cellulose, hemicelluloses, and gums, they differ in their solubility; the proportion of soluble:insoluble dietary fiber in flaxseed varies between 20:80 and 40:60 (Singh et al. 2011), whereas the soluble dietary fiber fraction in chia seed was reported to be much lower (the proportion of soluble:insoluble dietary fiber in chia seed was reported to be 5.33:94.67) (Din et al. 2021).

**FIGURE 1:**
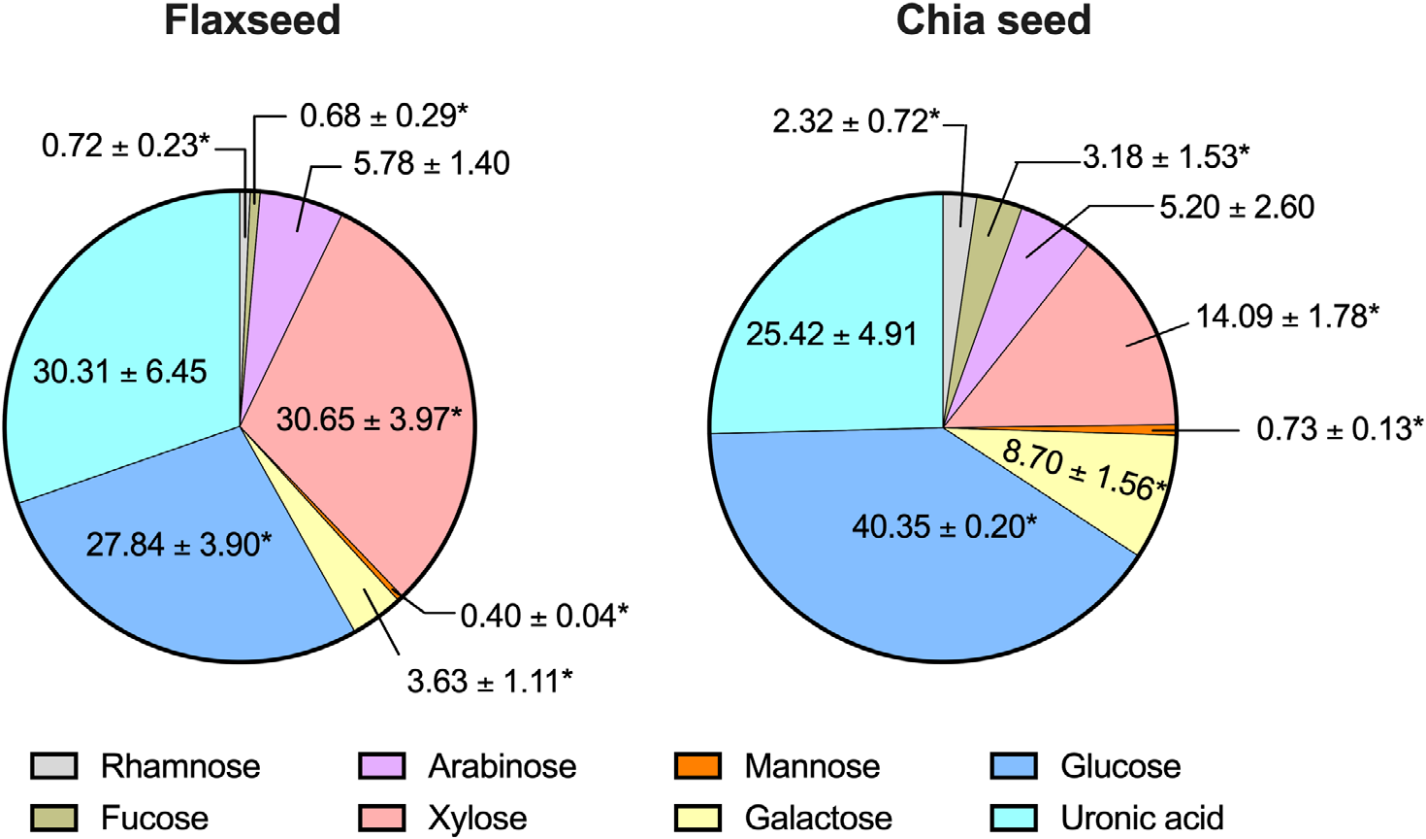
Monosaccharide compositions (%, weight basis) of dietary fibers of flaxseed and chia seed. The values are presented as mean ± standard deviation (*n* = 3). * indicates the significant differences between the monosaccharide moieties of the samples (two‐tailed student's *t*‐test, *p* < 0.05).

The monosaccharide compositions of dietary fibers of flaxseed and chia seed are shown in Figure 1. Flaxseed dietary fibers were primarily composed of xylose (30.65%), uronic acids (30.31%), and glucose (27.84%), with their respective ratios being closely comparable. In addition, lower concentrations of arabinose (5.78%) and galactose (3.63%) were detected, along with negligible amounts of rhamnose (0.72%), fucose (0.68%), and mannose (0.40%). The main insoluble and soluble dietary fiber fractions of flaxseed were reported to be cellulose and mucilage gums, respectively, and the mucilage gums compose of rhamnose (23.91%), glucose (19.98%), galactose (18.72%), xylose (13.50%), and other monosaccharide moieties (Safdar et al. 2019). Rhamnose and galactose content reported in this study are lower than the ones reported in the literature; however, the opposite was true for xylose. These discrepancies in the dietary fiber compositions could be attributed to possible uses of genetically and environmentally different flaxseed samples in different studies.

Conversely, glucose (40.35%) was found to be the primary component in chia seed dietary fiber, followed by uronic acid (25.42%) and xylose (14.09%). Additionally, lower amounts of galactose (8.70%), arabinose (5.20%), fucose (3.18%), rhamnose (2.32%), and mannose (0.73%) were also detected (Figure 1). These results align with previous reports where the water soluble fraction of chia seed (also known as chia seed gum) was demonstrated to compose of a tetrasaccharide repeating unit consisting of (1 → 4)‐β‐D‐xylopyranose‐(1 → 4)‐α‐D‐glucopyranose‐(1 → 4)‐β‐D‐xylopyranose as main chains and 4‐O‐methyl‐α‐D‐glucuronic acid as branching points at the O‐2 position of the β‐D‐xylopyranose residue (Lin et al. 1994). In addition, the presence of a significant amount of cellulose was reported in the insoluble fraction of chia seed polysaccharides (Din et al. 2021).

Comparatively, flaxseed dietary fibers were found to contain a significantly (*p* < 0.05) higher proportion of xylose than chia seed dietary fibers. On the other hand, the opposite was true in the cases of glucose, galactose, fucose, rhamnose, and mannose. No significant (*p* > 0.05) differences were observed between flaxseed and chia seed dietary fibers in terms of uronic acid and arabinose contents. Owing to their structural differences, the dietary fibers derived from chia seed and flaxseed are likely to exhibit distinct functional properties, potentially leading to divergent effects on the microbial community and its metabolic functions.

### 3.2 Short Chain Fatty Acid Production

**FIGURE 2:**
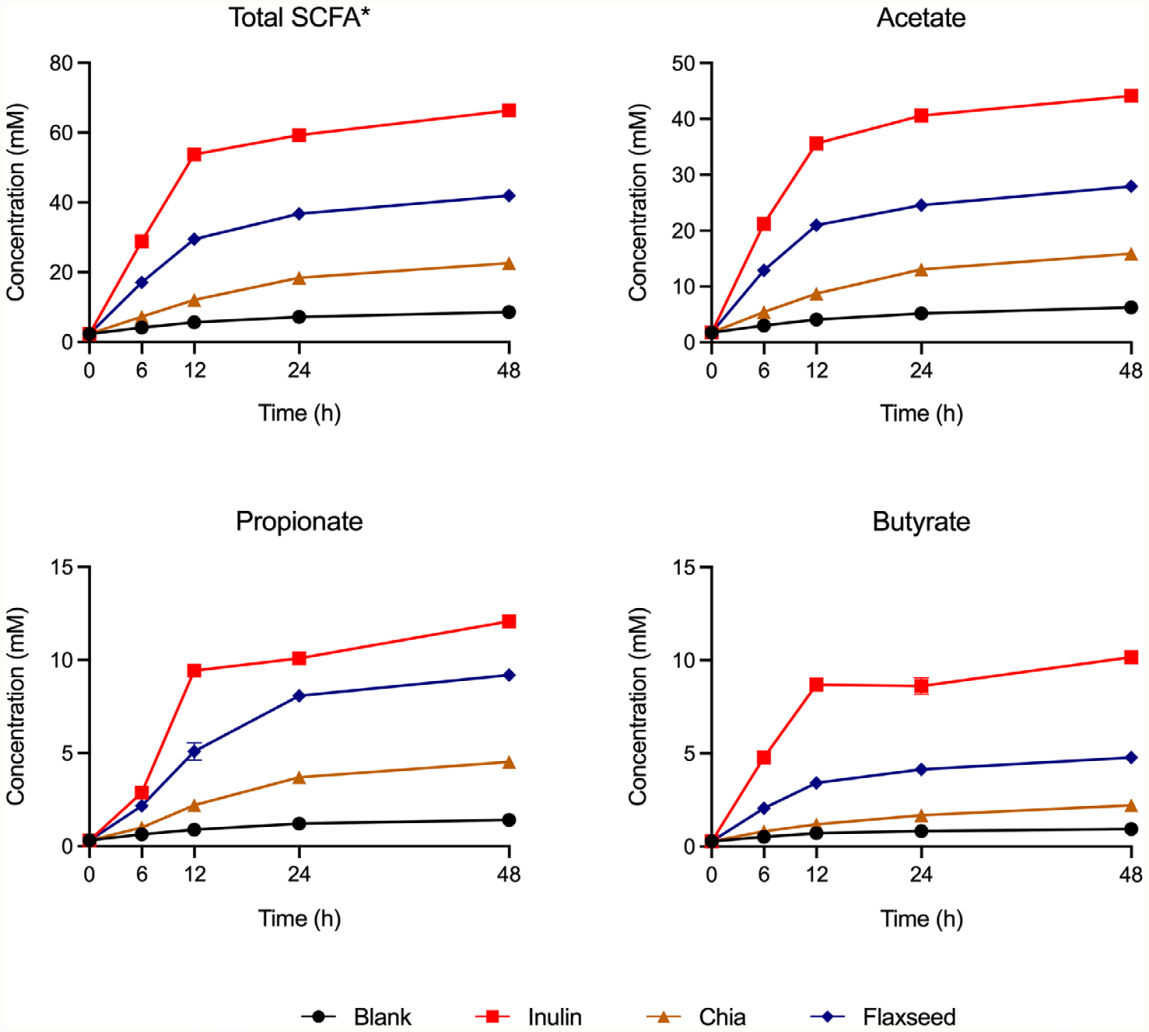
Short‐chain fatty acids (SCFAs—acetate, propionate, and butyrate) generated by fecal microbiota throughout in vitro fermentations of the dietary fibers of flaxseed and chia seed. Inulin was included as a fast‐fermenting, butyrate‐producing control. In the blank, there was no carbon source included (negative control). To calculate the total SCFA amounts, the quantities of acetate, propionate, and butyrate were summed. Error bars represent the standard error of the mean (*n* = 2).

Short‐chain fatty acids productions, namely acetate, butyrate, and propionate, by colonic microbiota as a result of fermentations of dietary fibers of chia seed and flaxseed were monitored for the course of a 48‐h fermentation (Figure 2). Both flaxseed and chia seed promoted SCFA production significantly more than the blank samples, but to a lesser extent than inulin, regardless of SCFA type, except for butyrate production from chia seed. In addition, flaxseed dietary fibers resulted in the generation of almost two‐fold more acetate, propionate, and butyrate than chia seed counterparts. This suggests that flaxseed dietary fibers are more readily fermented by the fecal microbiota compared to chia seed dietary fibers. As discussed above, previous studies showed that flaxseeds have higher soluble:insoluble dietary fiber proportions, compared to chia seeds (Singh et al. 2011; Din et al. 2021). Moreover, soluble dietary fibers are known to be fermented by the gut microbiota more rapidly than their insoluble counterparts (Hu et al. 2023; Wang et al. 2019; Tao et al. 2019). Therefore, the higher SCFA production by fecal microbiota observed in response to flaxseed dietary fibers compared to chia seed dietary fibers in this study may be attributed to the higher soluble‐to‐insoluble fiber ratio in flaxseed.

SCFAs that have various physiological functions in the human body are the most abundant microbial metabolites produced in the colon. For example, propionate is involved in the process of gluconeogenesis in the liver as a precursor. It is widely known that butyrate serves as an energy source for colon cells (Mann et al. 2024). Studies have shown that almost 95% of the butyrate produced by colonic microbiota is consumed within the colon. This high level of local utilization allows butyrate to play a crucial role in maintaining intestinal barrier integrity and regulating the inflammatory response (Zhang et al. 2021). Similarly, acetate, produced by bacterial fermentation in the colon, enters the bloodstream and performs various biological functions in conjunction with acetate released from body tissues and organs (Mann et al. 2024). In the liver, acetate is used as an energy source and is of great importance to the body, as it serves as a substrate for the synthesis of cholesterol and long‐chain fatty acids. Thus, stimulation of SCFAs by dietary fibers is often desired. The data obtained from this study suggests that flaxseed should be preferred over chia seed for the design of foods that target higher SCFAs generation in the colon.

### 3.3 Microbiota Responses to Chia and Flaxseed Dietary Fibers

Although in vitro fecal fermentation analyses were performed up to 48 h, microbial community analyses were done using the samples collected at the 24‐h time points. This is due to the fact that microbial SCFA generated during the fermentation reached their maximum values at 24‐h time points, and then not many changes were observed (almost remained in a plateau state) (Figure 2), suggesting that the majority of the microbial activity occurred in the first 24 h of the fermentation.

#### 3.3.1 Overall Structures of the Fecal Microbiota Communities

**FIGURE 3:**
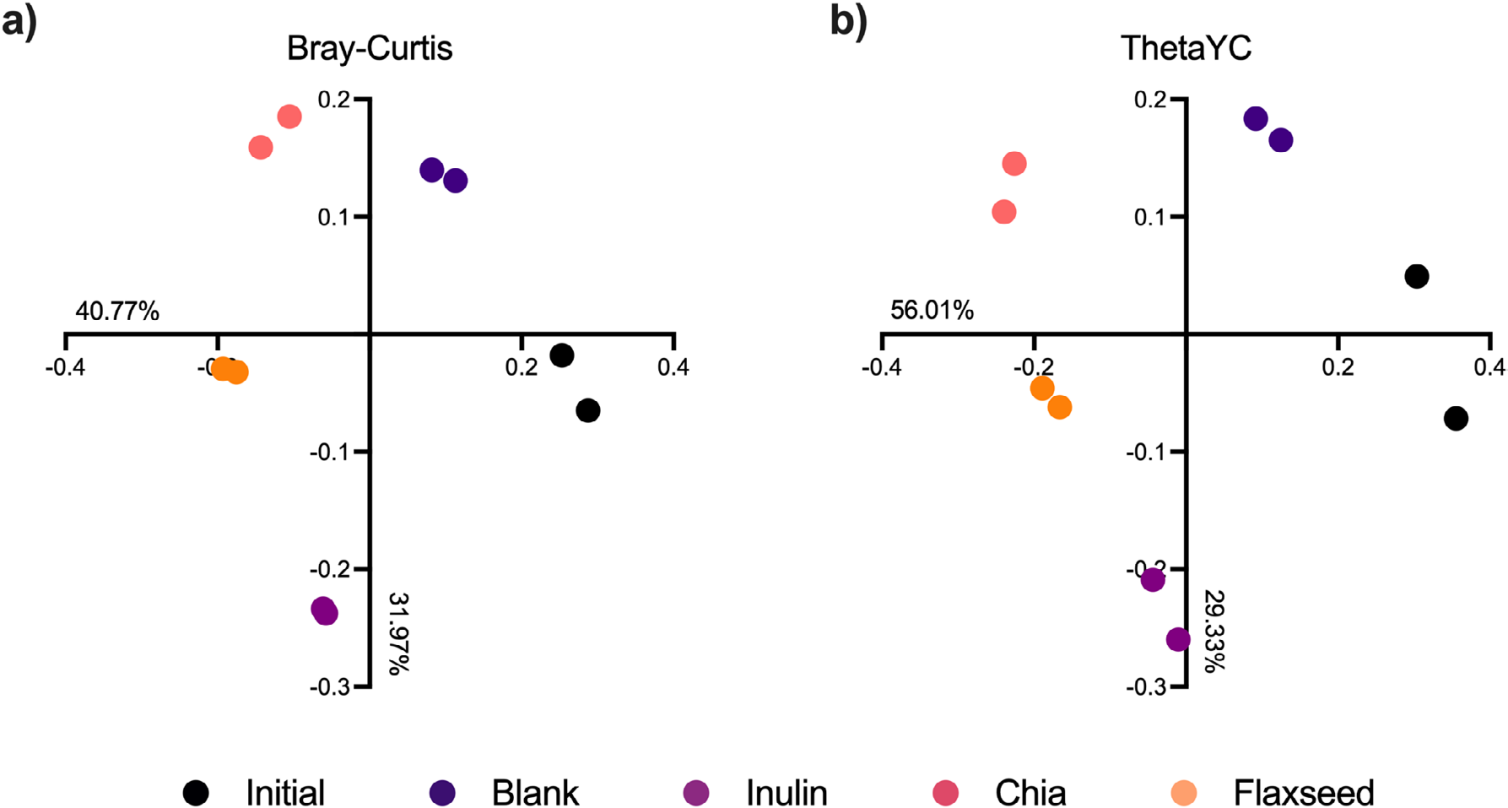
Principal coordinate analysis (PCoA) of (a) Bray–Curtis and (b) ThetaYC dissimilarity of microbiota communities based on the relative abundances of OTUs at a 97% identity level. Inulin was included as a fast‐fermenting, butyrate‐producing control. In the blank, there was no carbon source included (negative control).

Overall structures of the fecal microbiota communities were compared using the Bray–Curtis and ThetaYC dissimilarity tests based on the relative abundances of OTUs at a 97% similarity level (Figure 3a,b). Microbial communities of each sample were clustered accordingly, suggesting that the fiber type had a clear impact on their overall structures. Analysis of molecular variance (AMOVA) confirmed the significant (*p* < 0.001) effect of fiber type on microbial communities. Fiber type accounted for ~73% (for Bray–Curtis dissimilarity test) and ~85% (for ThetaYC dissimilarity test) of the total variation. Although microbial communities of each sample were clustered accordingly and fiber type was found to have a significant effect on the overall structures of microbial communities, the multiple comparisons test revealed no significant (AMOVA, *p* > 0.05) differences between chia and flaxseed communities. This could be attributed to the small sample set included in the study.

#### 3.3.2 α‐Diversity of the Microbial Communities

**FIGURE 4:**
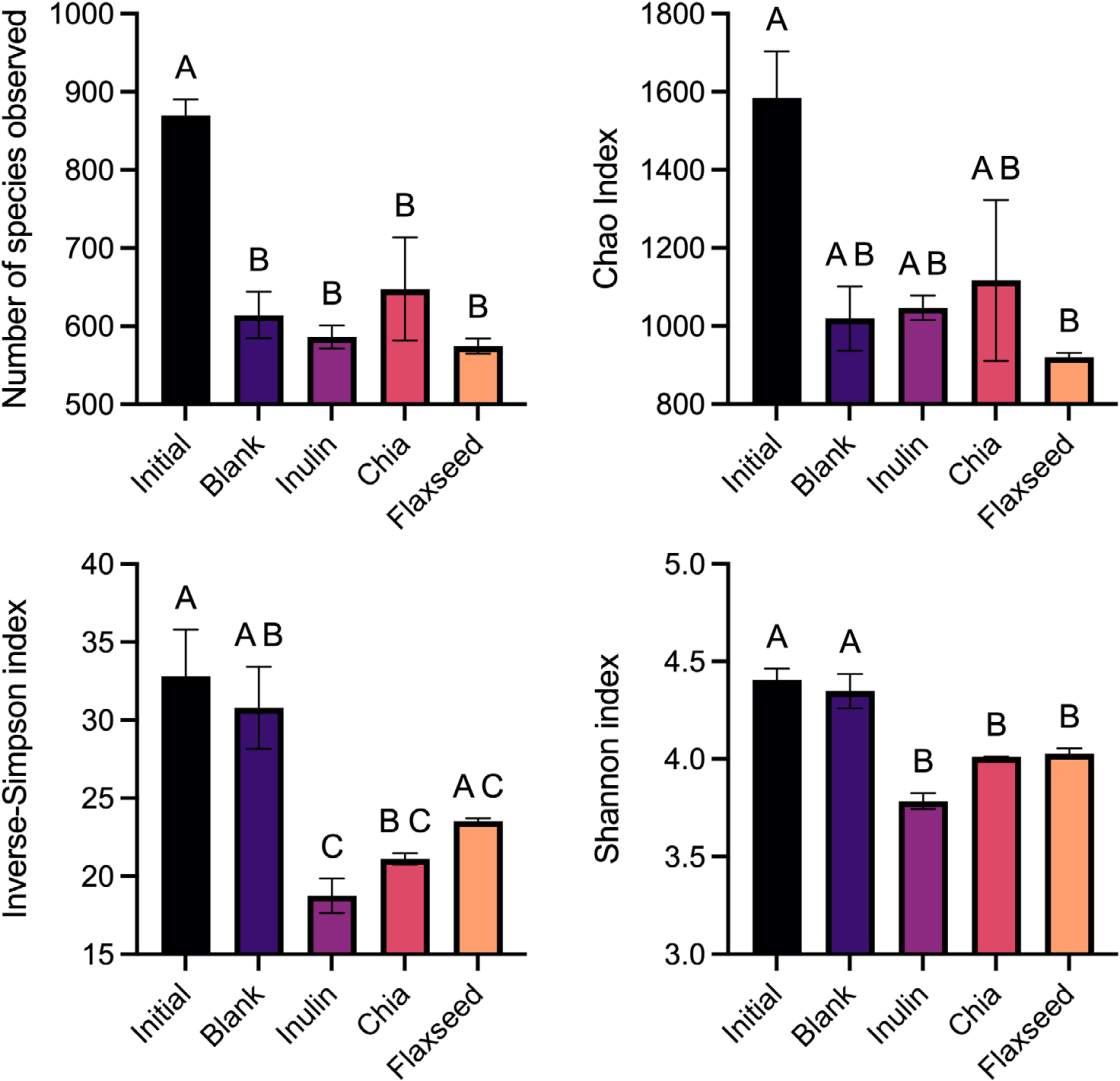
α‐Diversity (richness and/or evenness) of the microbial communities as measured by the number of species observed (NSO), Chao, inverse‐Simpson, and Shannon indices. Error bars represent the standard error of the mean (*n* = 2). Significant differences in mean values are denoted by different letters (*p* < 0.05, Tukey's multiple comparison test). Inulin was included as a fast‐fermenting, butyrate‐producing control. In the blank, there was no carbon source included (negative control).

The number of species observed (NSO), Chao, inverse‐Simpson, and Shannon index values were measured to determine the α‐diversity (richness and/or evenness) of the microbial communities. NSO and Chao index values account for species richness, whereas inverse‐Simpson and Shannon index values capture both species richness and evenness. Higher NSO or Chao index values mean the presence of a higher number of microbial species. Higher Simpson or Shannon index values indicate the presence of a high number of microbial species and their abundance is similar (Tuncil, Nakatsu, et al. 2017). Compared to time 0 (initial), significant (*p* < 0.05) decreases were observed in NSO values of all the samples (Figure 4). This was an expected result as in vitro fermentation tubes may not provide suitable niches for some of the gut microbiota, causing them to lose their activity during the fermentation. Similarly, decreases in the Chao index values of samples were observed compared to initial samples, but only significant differences were observed between microbial communities of initial and flaxseed dietary fiber (Figure 4). The discrepancies between NSO and Chao index values could be attributed to different mathematical formulations used in their calculations. Similarly, inverse‐Simpson and Shannon indices of samples decreased over the fermentation, except for the fact that the inverse‐Simpson index value of the microbial community belonging to the flaxseed group did not significantly (*p* > 0.05) differ from that of initial (Figure 4). This suggests that flaxseed, but not chia seed, dietary fibers have the ability to retain the α‐diversity of the microbial communities. This could be attributed to the structural differences observed in the dietary fibers of flaxseed and chia seed. However, the same trend could not be observed in their Shannon index values.

#### 3.3.3 Changes in the Microbial Composition at the OTU Level

**FIGURE 5:**
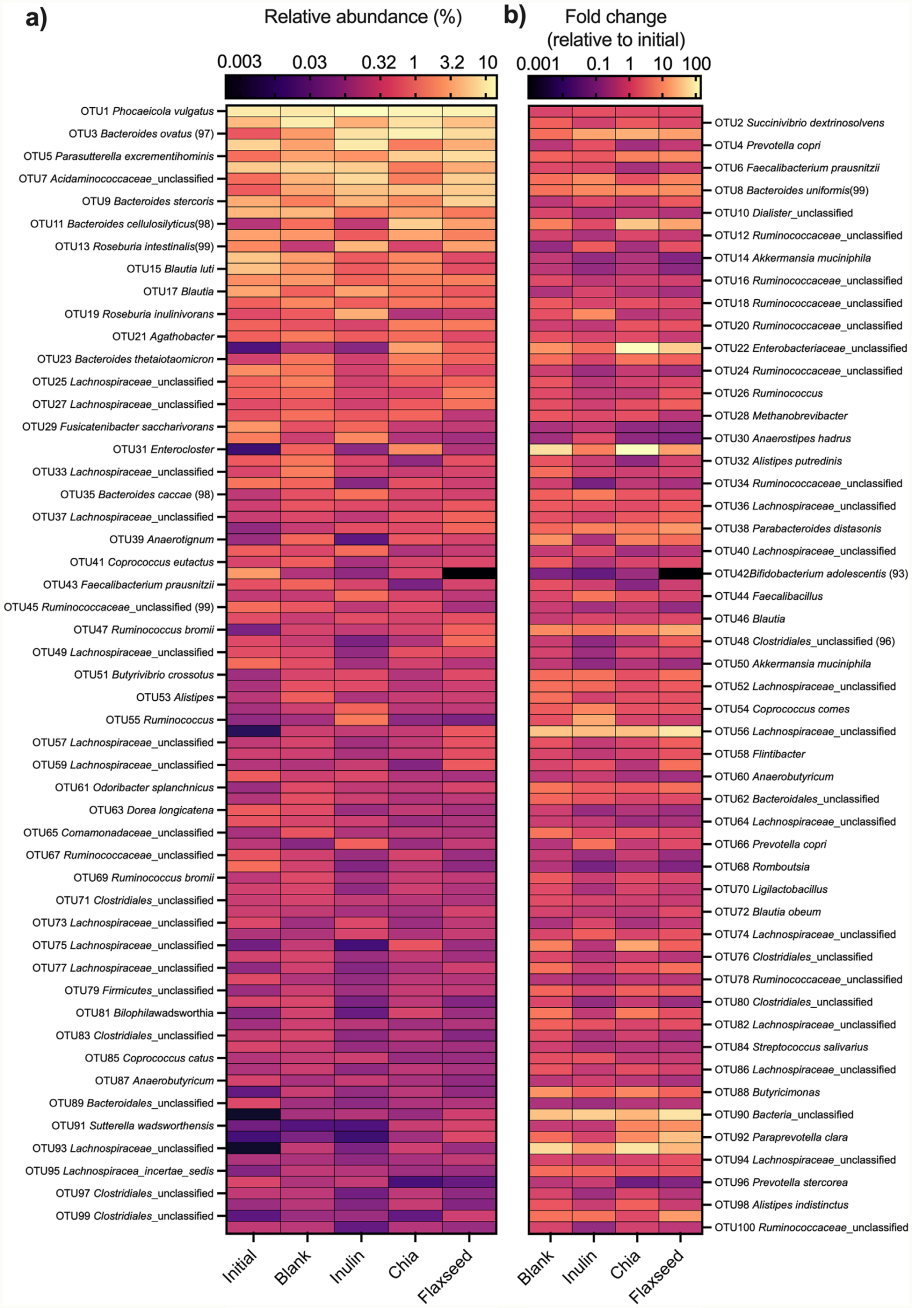
(a) The relative abundances (as a percentage of sequencing reads) of the most abundant 100 taxa prior to and following 24 h of in vitro fecal fermentation. (b) Fold‐changes in the relative abundances of each OTU relative to initial time point (time 0—before fermentation). Due to space constraints in the figure, odd‐numbered OTUs are labeled in the left heat map, and even‐numbered OTUs are labeled in the right heat map. Inulin was included as a fast‐fermenting, butyrate‐producing control. In the blank, there was no carbon source included (negative control).

**FIGURE 6:**
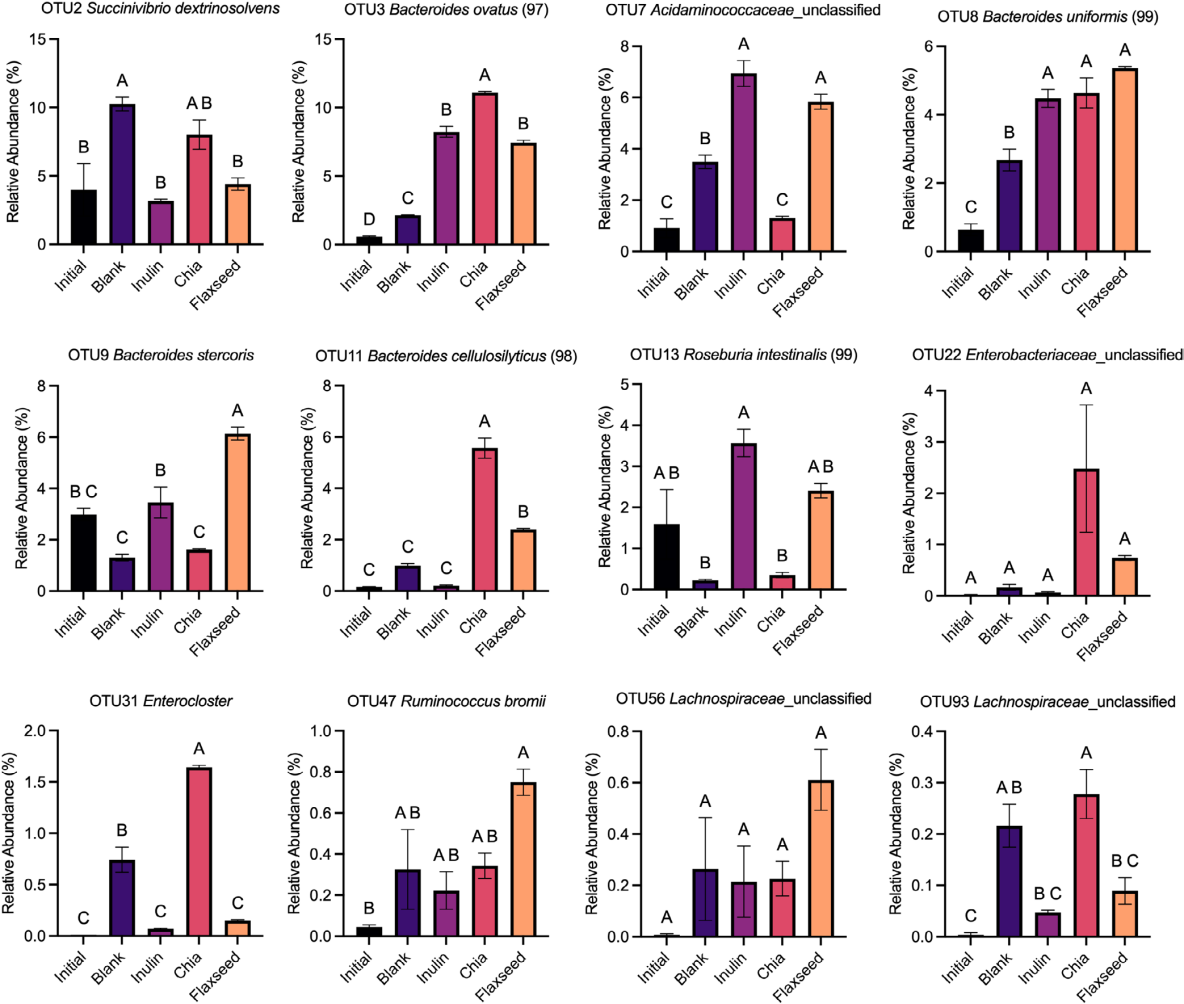
Relative abundances (as a percentage of sequencing reads) of some OTUs before and after 24 h of in vitro fermentation. Error bars represent the standard error of the mean (*n* = 2). Significant differences in mean values are denoted by different letters (*p* < 0.05, Tukey's multiple comparison test). Inulin was included as a fast‐fermenting, butyrate‐producing control. In the blank, there was no carbon source included (negative control).

The effects of the dietary fibers of flaxseed and chia seed on the compositions of fermenting microbiota were investigated at the OTU level. The relative abundances of the 100 most abundant microbial OTUs and the fold changes (relative to time 0) in their relative abundances after 24 h of fermentation were illustrated in Figure 5a,b, respectively. The 100 most abundant microbial OTUs were calculated to constitute > 84% of the total bacterial community. Additionally, the relative abundances of the most responsive microbial taxa were also graphed in Figure 6 along with statistical analyses. The results revealed that the fermentations of dietary fibers of flaxseed and chia seed differentially impacted the fecal microbiota compositions. Specifically, compared to time 0 (0.92%), fermentation of flaxseed dietary fibers significantly (*p* < 0.05) increased the relative abundance of *Acidaminococcaceae‐related* OTU (OTU7) to 5.84%, causing 6.4‐fold increases; however, no significant (*p* > 0.05) change was observed in the case of chia seed dietary fibers (Figure 6). Similarly, a significant (*p* < 0.05) increase was observed in the relative abundance of OTU9 *Bacteroides stercoris* with the fermentation of dietary fibers of flaxseed (from 2.99% to 6.14%), but not with that of chia seed (from 2.99% to 1.62%) (Figure 6). These promotions in the relative abundances of *Acidaminococcaceae* and *B.* *stercoris‐related* OTUs are most likely to be one of the main reasons why fermentation of dietary fibers of flaxseed resulted in higher amounts of propionate compared to that of chia seed, because both *Acidaminococcaceae* members and *B. stercoris* are known to have the ability to generate propionate in the human colon as a result of fermentations of dietary constituents (Louis and Flint 2017; Shagaleeva et al. 2023). Conversely, at the end of the fermentation, the relative abundances of OTU3 *Bacteroides ovatus* and OTU11 *B. cellulosilyticus* were significantly (*p* < 0.05) higher in the chia seed dietary fiber group than in the flaxseed dietary fiber group (Figure 6). *Bacteroides* species are important members of the human colon that have a great ability to utilize dietary fibers (Koropatkin et al. 2012). Indeed, compared to time 0, both flaxseed and chia seed groups significantly promoted the *Bacteroides‐related* OTUs (OTU3, OTU8, and OTU11), which could be attributed to the overall great dietary fiber utilization abilities of *Bacteroides* members. However, the degrees of *Bacteroides* promotions were fiber‐type dependent. This could be attributed to the different chemical compositions of dietary fibers of chia and flaxseed (Figure 1), as dietary fiber fermentations by different *Bacteroides* species are tightly tuned to their chemical structures (Tuncil, Xiao, et al. 2017).

In addition, fermentations of dietary fibers of chia seed and flaxseed resulted in 5.9‐ and 7.9‐fold increases in the relative abundances of OTU5 *Parasutterella excrementihominis* , respectively, compared to time 0 (its initial relative abundance was 0.97%, while at the end of the fermentation its relative abundance was 5.97%, and 7.61% in chia seed and flaxseed group, respectively) (Figure 5). *Parasutterella* genus has been recognized as a member of the healthy microbiome of the human gastrointestinal tract. *P. excrementihominis* has also been associated with various host health outcomes, including reduced risk of inflammation and obesity (Galley et al. 2024; Ju et al. 2019; Li et al. 2025).

Compared to time 0, fermentation of flaxseed dietary fibers did not cause any significant (*p* > 0.05) changes in the relative abundances of *Enterocloster‐related* OTU (OTU31) (Figure 6), some of whose members (i.e., *E. bolteae*) are known as gut pathogens and associated with dysbiosis and human diseases such as autism and inflammatory bowel diseases (Magdy Wasfy et al. 2023). However, fermentation of chia seed dietary fibers resulted in a dramatic increase in its relative abundance (164‐folds increases; relative abundance was increased from 0.01% to 1.64%). These collectively suggest that flaxseed, but not chia seed, dietary fibers have the ability to suppress the growth of pathogenic microorganisms in the colon.

Although fold‐change results illustrated in Figure 5b showed that the fermentation of chia seed dietary fibers resulted in almost 138‐fold increases in the relative abundances of *Enterobacteriaceae* related OTU (OTU22), some of its members are known as gut pathogens and are associated with dysbiosis and inflammatory bowel diseases (Baldelli et al. 2021), this increase was not significant (*p* > 0.05), and no significant (*p* > 0.05) differences were observed in the relative abundances of this OTU between any of the samples (Figure 6).

## 4 Conclusions

In this study, the effects of chia and flaxseed dietary fibers were comparatively evaluated for the first time. Our results revealed that, compared to dietary fibers of chia seed, that of flaxseed is likely to be more readily fermentable by the fecal microbiota. Additionally, fermentations of the dietary fibers of flaxseed and chia seed differentially impacted the fecal microbiota compositions at the OTU level; specifically, significant increases in the relative abundances of *Acidaminococcaceae* and *B. stercoris* related OTUs, which are known to have the ability to generate propionate, a healthy microbial metabolite, were observed in the case of flaxseed, but not chia seed. Accordingly, significantly higher propionate formation was observed in flaxseed, compared to chia seed samples. Interestingly, fermentation of flaxseed dietary fibers did not change the relative abundance of *Enterocloster* related OTU (OTU31) which harbors some pathogenic bacteria such as *E. bolteae*; however, dramatic increases in its relative abundance were observed in the case of chia seed dietary fibers. This suggests that flaxseed, but not chia seed, dietary fibers may have the ability to suppress the growth of some pathogenic bacteria in the colon. Finally, fermentations of both flaxseed and chia seed dietary fibers were found to promote *Bacteroides* related OTUs (i.e., OTU3 *B. ovatus* and OTU11 *B. cellulosilyticus* ); however, the degree of promotion was found to be seed‐type dependent, which can be attributed to their different compositional features. Overall, this study clearly demonstrates that the effects of flaxseed and chia seed dietary fibers on the colonic microbiota vary, with flaxseed fibers being more easily fermented and more effective in promoting beneficial microbial growth. Therefore, flaxseed (and its dietary fibers) could potentially be used for designing functional (healthier) food recipes targeting colonic health. Future research should aim to validate these findings through in vivo studies and clinical trials.

## Author Contributions

**Seda Arioglu‐Tuncil:** conceptualization (equal), data curation (equal), formal analysis (equal), funding acquisition (equal), investigation (equal), methodology (equal), project administration (equal), resources (equal), software (equal), supervision (equal), validation (equal), visualization (equal), writing – original draft (equal), writing – review and editing (equal).

## Conflicts of Interest

The author declares no conflicts of interest.

## Data Availability

The data that support the findings of this study are available on request from the corresponding author.
